# Enhancing folic acid metabolism suppresses defects associated with loss of *Drosophila* mitofusin

**DOI:** 10.1038/s41419-019-1496-2

**Published:** 2019-03-25

**Authors:** Juan Garrido-Maraver, Ivana Celardo, Ana C. Costa, Susann Lehmann, Samantha H. Y. Loh, L. Miguel Martins

**Affiliations:** MRC Toxicology Unit, University of Cambridge, Lancaster Road, Leicester, LE1 9HN UK

## Abstract

Mutations in the mitochondrial GTPase mitofusin 2 (MFN2) cause Charcot-Marie-Tooth disease type 2 (CMT2A), a form of peripheral neuropathy that compromises axonal function. Mitofusins promote mitochondrial fusion and regulate mitochondrial dynamics. They are also reported to be involved in forming contacts between mitochondria and the endoplasmic reticulum. The fruit fly, *Drosophila melanogaster*, is a powerful tool to model human neurodegenerative diseases, including CMT2A. Here, we have downregulated the expression of the *Drosophila* mitofusin (*dMfn* RNAi) in adult flies and showed that this activates mitochondrial retrograde signalling and is associated with an upregulation of genes involved in folic acid (FA) metabolism. Additionally, we demonstrated that pharmacological and genetic interventions designed to increase the FA metabolism pathway suppresses the phenotype of the dMfn RNAi flies. We conclude that strategies to increase FA metabolism may ameliorate diseases, such as peripheral neuropathies, that are associated with loss of mitochondrial function. A video abstract for this article is available at https://youtu.be/fs1G-QRo6xI.

## Introduction

Chartcot-Marie-Tooth (CMT) disease is the most common inherited neuromuscular disorder^1^, with no cure or treatment available. This peripheral neuropathy results from damage to neurons that transmit information from the brain and spinal cord to other parts of the body. Individuals with CMT suffer from muscle weakness and atrophy as well as mild sensory loss. There are two main types of CMT: type 1, where the damage occurs to myelin sheaths that surround the axons of neurons (CMT1) and type 2, where the damage is to the neuron itself (CMT2). Mutations in mitofusin 2 (*MFN2*) have been found to be one of the most common causes of CMT2, called CMT2a^2^.

Mitofusins are GTP-hydrolysing enzymes that promote mitochondrial fusion. They are important to ensure the dynamic balance between fusion and fission that determines mitochondria morphology . This dynamic balance also regulates mitochondrial health, since fission is crucial for recycling defective mitochondria through a process called mitophagy (reviewed in^3^).

Mitofusins can also act as organelle bridges. Mfn2 was reported to link mitochondria to the endoplasmic reticulum (ER)^4^ and the downregulation of *Drosophila* mitofusin (*dMfn*, also known as *Marf*) reduced contacts between the ER and mitochondria^5^.

The fruit fly, *Drosophila melanogaster*, is a powerful tool to study human neurodegenerative diseases (reviewed in^6^). Moreover, pharmacological approaches can be used to test therapeutic candidates in flies. Drugs can be incorporated in the food and readily delivered. Since flies lack a stringent blood-brain barrier, these drugs can easily access the nervous system (reviewed in^7^).

Previously, it was reported that the ubiquitous downregulation of *dMfn* in *Drosophila* is lethal at the larval stage^8^ and causes cellular toxicity by activating ER stress^9^. Here, we employed a strategy that enabled the generation of adult flies with ubiquitous downregulation of *dMfn* and explored the consequences of its loss. We show the downregulation of *dMfn* in adult flies compromises mitochondrial function and slows down the axonal transport of mitochondria in wing sensory neurons.

We used an exploratory analysis of the toxic consequences of downregulating *dMfn* in adult flies. We uncovered an alteration of folic acid (FA) metabolism transcripts in flies with downregulated *dMfn*. We show that both dietary and genetic interventions to enhance FA metabolism partially supressed the defects observed in adult flies lacking *dMfn*. We conclude that strategies to enhance FA metabolism may prevent or delay the axonal defects in *MFN2*-linked CMT2a and other peripheral neuropathies associated with mitochondrial dysfunction.

## Results

### Reducing the expression of *dMfn* compromises motor function in adult flies

**Fig. 1:**
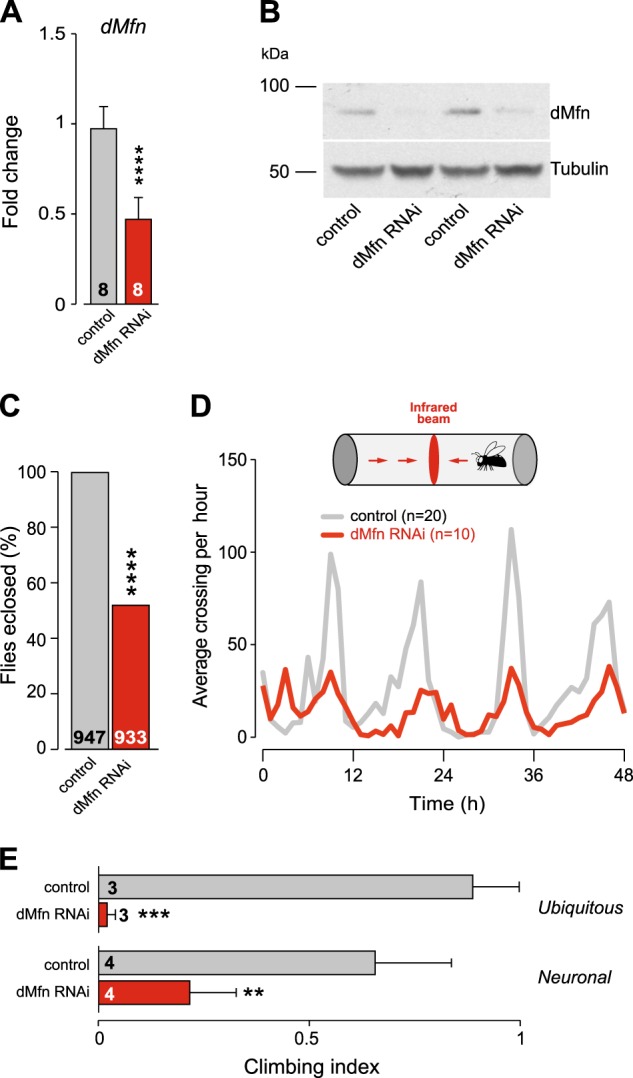
In vivo suppression of *dMfn* causes motor impairment in adult flies. **a** and **b** RNAi-mediated suppression of *dMfn*. **a** Expression levels of the *dMfn* transcript were measured by real-time qPCR (mean ± SD; asterisks, two-tailed unpaired *t*-test compared to control). **b** Analysis of dMfn protein levels. Whole-fly lysates were analysed using the indicated antibodies. **c** Eclosion defects following RNAi-mediated suppression of *dMfn* (asterisks, chi-square two-tailed, 95% confidence intervals). **d** Suppression of *dMfn* decreases locomotor activity. The number of flies tested is indicated for each genotype. **e** Motor impairment upon RNAi-mediated suppression of *dMfn*. Flies were tested using a standard climbing assay (mean ± SD; asterisks, one-way ANOVA with Dunnett’s multiple comparison test). Genotypes in (**a**–**e** (ubiquitous)): Control: *w; +; daGal4/+*, dMfn RNAi: *w; dMfn RNAi/+; daGal4/+*. (**e** (neuronal)): Control: *w; elavGal4/+; +*, dMfn RNAi: *w; elavGal4/dMfn RNAi; +*

The ubiquitous downregulation of *dMfn* by RNAi using the *tubulin*-Gal4 driver is lethal at the larval stage^8^. By using an alternative ubiquitous driver, *daughterless*-Gal4 (*da*Gal4), we generated adult *dMfn* RNAi flies where the *dMfn* transcript was downregulated by 50% (Fig. 1a). This also reduced dMfn protein levels (Fig. 1b) and enabled approximately 50% of the adult flies to hatch (Fig. 1c). We then investigated the basal locomotor activity in *dMfn* RNAi flies and found that the loss of *dMfn* resulted in locomotor defects (Fig. 1d). Additionally, both the ubiquitous or pan-neuronal downregulation of *dMfn* resulted in climbing defects (Fig. 1e).

### Downregulation of *dMfn* expression results in a loss of mitochondrial cristae and reduced mitochondria axonal transport

**Fig. 2:**
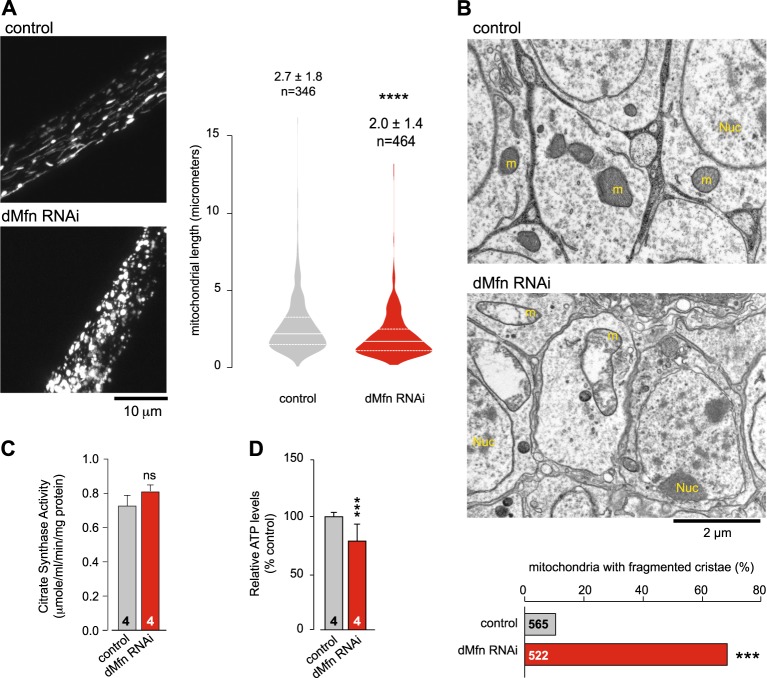
Suppression of *dMfn* causes a loss of mitochondrial cristae density. **a** RNAi-mediated suppression of *dMfn* results in mitochondrial fragmentation. Confocal analysis of mitoGFP in the larval mechanosensory axons. The quantification of mitochondrial length is shown as a combined violin and box plot (*p* value, two-tailed unpaired *t*-test compared to control). **b** The knockdown of *dMfn* causes defects in mitochondrial cristae. Ultrastructural analysis of the brains in adult *dMfn* RNAi flies (m, mitochondria; nu, cell nuclei). Asterisks, chi-square two-tailed, 95% confidence intervals. **c** The knockdown of *dMfn* does not affect overall mitochondrial mass. Mitochondrial mass was assessed by measuring the activity of the mitochondrial matrix enzyme citrate synthase in adults (ns, *p* > 0.05, two-tailed unpaired *t*-test compared to control). **d** The knockdown of *dMfn* causes a loss of ATP in adult flies (mean ± SD, asterisks, two-tailed unpaired *t*-test compared to control). Genotypes in **a** Control: *w; elavGal4/+; UASmitoGFP/+*, dMfn RNAi: *w; elavGal4/dMfn RNAi; UASmitoGFP/+*. **b**–**d** Control: *w; +; daGal4/+*, dMfn RNAi: *w; dMfn RNAi/+; daGal4/+*

**Fig. 3:**
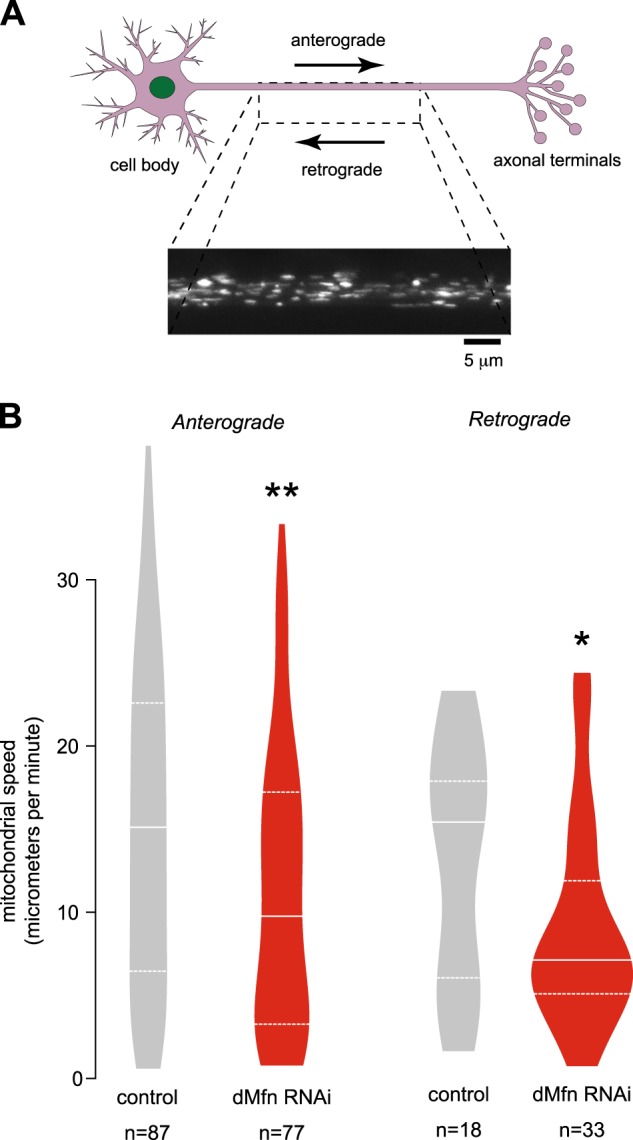
The knockdown of *dMfn* decreases axonal mitochondrial motility. **a** Representative image of a control wing nerve with mitoGFP expression and a scheme indicating the direction of anterograde and retrograde mitochondrial transport. **b** Decreased mitochondrial speed in *dMfn* RNAi wing sensory axons. Both anterograde and retrograde mitochondrial speed were measured for the indicated genotypes. Combined violin and box plot. (*n* is the number of moving mitochondria analysed, asterisks, two-tailed unpaired *t*-test compared to control). Genotypes: Control: *w; dprGal4/+; UASmitoGFP/+*, dMfn RNAi: *w; dprGal4/dMfn RNAi; UASmitoGFP/+*

We next performed an analysis of mitochondria in *dMfn* RNAi flies. A morphological analysis of the larval ventral nerve cord revealed fragmentation of mitochondria in the mechanosensory axons (Fig. 2a). Ultrastructural analysis of adult fly brains showed fragmented mitochondria cristae (Fig. 2b). To determine if the ultrastructural defects observed upon depletion of *dMfn* were associated with a decrease in mitochondrial density (MD), we measured the levels of the mitochondrial matrix enzyme citrate synthase^10^, an indirect measurement of MD, in adult flies. Citrate synthase levels in *dMfn* RNAi flies were not significantly altered compared to controls (Fig. 2c), indicating that the loss of mitochondrial cristae following depletion of *dMfn* is not accompanied by a generalised loss of mitochondrial mass. Mitochondria are responsible for the generation of the majority of cellular ATP, a source of energy for axonal mitochondrial transport (reviewed in^11^). We also observed a significantly lower level of ATP in *dMfn* RNAi flies compared to the control (Fig. 2d). Moreover, MFN2 mutations present in CMT2 patients cause defects in mitochondrial transport in cultured neurons^12^. We therefore assessed the effect of lowering *dMfn* expression on the axonal transport of mitochondria to (retrograde) and away (anterograde) from the cell nucleus in wing sensory neurons (Vagnoni et al., 2016). We observed that lowering the expression of *dMfn* results in decreased speeds for both anterograde and retrograde transport of mitochondria (Fig. 3).

**Fig. 4:**
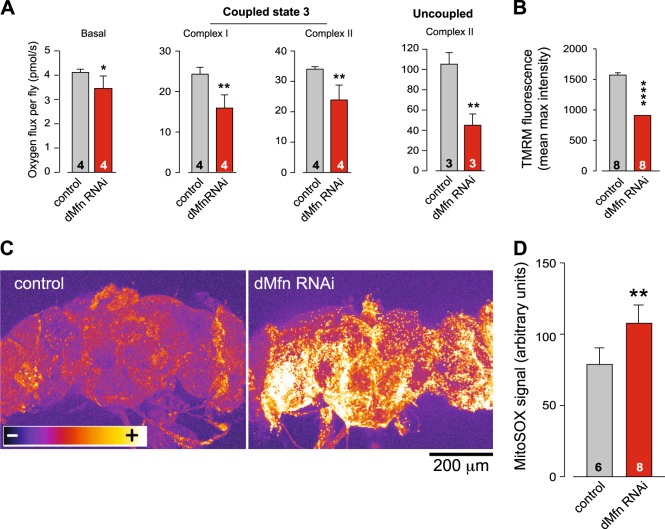
The knockdown of *dMfn* reduces mitochondria respiration and leads to an increase in reactive oxygen species. **a** Decreased respiration in *dMfn* RNAi flies. Activity was measured by high-resolution respirometry in 3-day-old flies. Data are shown as the means ± SD (n ≥ 4 in each genotype); asterisks, two-tailed unpaired *t*-test. **b** Loss of Δψm following RNAi-mediated suppression of *dMfn*. The data are shown as the mean ± SD; asterisks, two-tailed unpaired *t*-test. **c** and **d** Increased mitochondrial ROS production in *dMfn* RNAi flies. **c** Representative images of adult fly brains stained with MitoSOX using false colour rendering. **d** Quantification of mitoSOX signal following the RNAi-mediated suppression of *dMfn* (mean ± SD; asterisks, two-tailed unpaired *t*-test). Genotypes: **a**-**d** Control: *w; +; daGal4/+*, dMfn RNAi: *w; dMfn RNAi/+; daGal4/+*

We next focused on the functional status of mitochondria in adult flies with decreased expression of *dMfn*. High-resolution respirometry analysis revealed a significant decrease in respiration rates following suppression of *dMfn* (Fig. 4a). Furthermore, we detected a loss of mitochondrial membrane potential (Δψm) (Fig. 4b) and an increase in the levels of reactive oxygen species (ROS) (Fig. 4c, d) in the brains of adult flies with decreased expression of *dMfn*. These results, together with the loss of mitochondrial cristae integrity (Fig. 2b), suggest that the suppression of *dMfn* does not affect the overall quantity of mitochondria but does compromise their function.

### Decreased expression of *dMfn* is associated with the activation of ATF4-dependent targets associated with folic acid metabolism

**Fig. 5:**
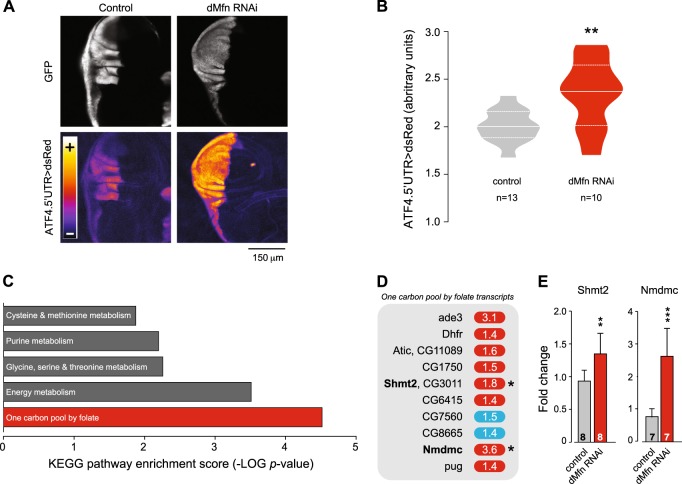
The knockdown of *dMfn* is associated with an enhanced expression of one-carbon metabolism transcripts. **a** and **b** The RNAi-mediated suppression of *dMfn* activates *dATF4*. RNAi *dMfn* was targeted at the posterior compartment of the larval wing disc using *hh*Gal4 recombined with a GFP reporter. **a** Representative confocal images showing the enhanced in vivo expression of the ATF4.5’UTR > dsRed reporter (lower right panel) following the RNAi-mediated suppression of *dMfn* (posterior to the left and anterior to the right). **b** Quantitative analysis of dsRed fluorescence in the wing discs of the indicated genotypes. Combined violin and box plot (asterisks, two-tailed unpaired *t*-test). **c** and **d** Enhanced expression of one-carbon pool by folate metabolism transcripts following the RNAi-mediated suppression of *dMfn*. **c** Top pathways, comprising functionally related genes identified using Partek Pathway Analysis software are shown. *P*-values were calculated using Fisher’s exact test. See also Supplementary Table 3. **d** Individual transcripts belonging to the one-carbon pool by folate metabolism enriched following the RNAi mediated suppression of *dMfn*. Red and blue correspond to, respectively, upregulated and downregulated transcripts above a statistical threshold (FDR ≤ 5%). Asterisks indicate transcripts previously shown to be controlled by *dATF4*. **e** *dMfn* RNAi flies show an upregulation of mitochondrial *Shmt2* and *Nmdmc* (mean ± SD; asterisks, two-tailed unpaired *t*-test). Genotypes: **a**–**b** Control: *w; ATF4.5′ UTR* > *dsRed/+; hhGal4,UASGFP/+*, dMfnRNAi: *w; ATF4.5’UTR* > *dsRed/dMfnRNAi; hhGal4,UASGFP/+*. **c**–**e** Control: *w; +; daGal4/+*, dMfn RNAi: *w; dMfn RNAi/+; daGal4/+*

Debattisti and colleagues showed that loss of *dMfn* activates ER stress^9^. We have recently demonstrated that ER stress linked to mitochondrial dysfunction causes the activation of dATF4, a transcription factor involved in cellular adaptation to stress^13^. To determine if a decrease in *dMfn* could affect *dATF4* expression, we measured the levels of activation of this transcription factor in *dMfn* RNAi tissues using a reporter for *dATF4* activation^14^. This analysis showed that the decreased expression of *dMfn* led to a marked activation of *dATF4* (Fig. 5a, b). The response to ER stress involves a generalised decrease in protein translation mediated by PERK and a concomitant activation of ATF4-dependent transcriptional programmes designed to induce a cellular adaptation to stress (reviewed in^15^). To identify such transcriptional programmes in *dMfn* RNAi flies, we took an unbiased approach by using microarray analysis. The analysis of transcriptional changes in *dMfn* RNAi flies (Supplementary Table 1) showed a significant (FDR step-up ≤ 0.05) upregulation of 275 transcripts with fold-change ≥ 1.6 (Supplementary Table 2). Next, a pathway analysis of the upregulated transcripts showed that the decrease in *dMfn* expression causes an activation of genes involved in FA metabolism (Fig. 5c, d and Supplementary Table 3), including mitochondrial serine hydroxymethyl transferase (*Shmt2*) and mitochondrial NAD-dependent methylenetetrahydrofolate dehydrogenase (*Nmdmc*), two transcriptional targets of *dATF4* that code for mitochondrial proteins^13^. The increased levels of *Shmt2* and *Nmdmc* transcripts following *dMfn* RNAi were confirmed in adult flies using real-time qPCR (Fig. 5e).

### A diet supplemented with folic acid suppresses mitochondrial dysfunction

Folic acid has been historically used to treat anaemia during pregnancy. It functions as a carbon donor in metabolic reactions, including those involved in the synthesis of nucleotides from purine precursors (reviewed in^16^), and it has been shown to counteract mitochondrial dysfunction in the *Drosophila* central nervous system^17^.

Our data show that in the context of decreased levels of *dMfn*, flies attempt to compensate for mitochondrial stress by inducing the expression of FA metabolism genes. We therefore tested if enhancing this metabolic pathway by increasing the bioavailability of FA could rescue cellular defects associated with the loss of *dMfn*.

**Fig. 6:**
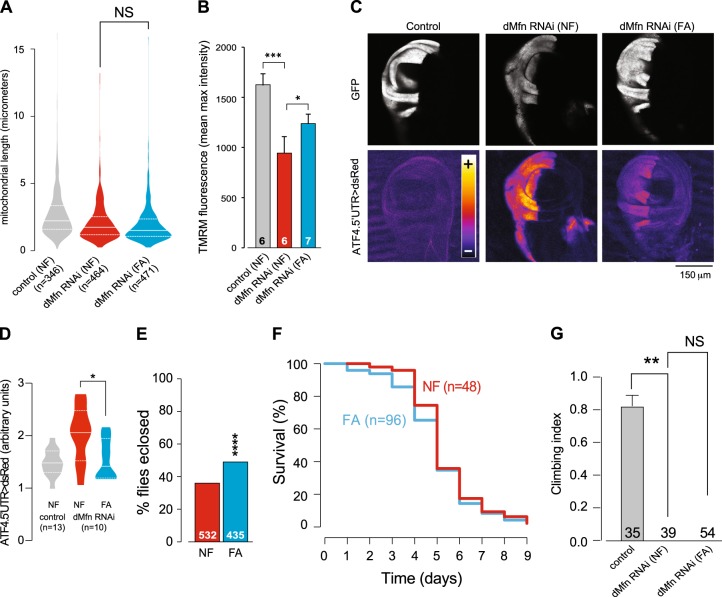
Dietary supplementation with FA suppresses defects associated with RNAi-mediated suppression of *dMfn*. Dietary supplementation with FA does not affect mitochondrial fragmentation in *dMfn* RNAi flies (**a**) but partially reverses the loss of Δψm (**b**) (mean ± SD; asterisks, one-way ANOVA with Bonferroni’s multiple comparison test). **c**, **d** Dietary supplementation with FA suppresses the increased expression of *dATF4* in *dMfn* RNAi flies. Representative confocal images and quantitative analysis of dsRed fluorescence in the indicated wing discs are shown (asterisk, two-tailed unpaired *t*-test). **e** Decreased eclosion defects in *dMfn* RNAi flies, following dietary supplementation with FA (asterisks, chi-square two-tailed, 95% confidence intervals). **f** An FA-supplemented diet does not modify lifespan upon RNAi-mediated suppression of *dMfn*. Fly viability was scored over a period of 10 days. **g** An FA supplemented diet does not rescue the locomotor defects of *dMfn* RNAi flies. Flies were tested using a standard climbing assay (mean ± SD; asterisks, one-way ANOVA with Dunnett’s multiple comparison test). *NF* normal food; *FA* folic acid supplemented food. Genotypes: (**a**) Control: *w; elavGal4/+; UASmitoGFP/+*, dMfn RNAi: *w; elavGal4/dMfn RNAi; UASmitoGFP/+*, **c**–**d** Control: *w; ATF4 5′ UTR DsRed/+; hhGal4,UASGFP/+*, dMfn RNAi: *w; ATF4.5′ UTR* > *dsRed/dMfn RNAi; hhGal4,UASGFP/+*, **e**–**f** *w; dMfn RNAi/+; daGal4/+* and (**b** and **g**) Control: *w; +; daGal4/+*, dMfn RNAi: *w; dMfn RNAi/+; daGal4/+*. Datasets in (**a**), labelled control (NF) and dMfn RNAi (NF) are also used in Fig. 2a. Dataset in (**b**) labelled dMfn RNAi (NF) is also used in Fig. 7a, labelled dMfn RNAi. Dataset in (**d**) labelled control (NF) and dMfn RNAi (NF) are also used in Fig. 5b

Maintaining *dMfn* RNAi flies on an FA-supplemented diet during embryonic development did not alter mitochondrial fragmentation in larval mechanosensory axons (Fig. 6a) but was able to suppress loss of Δψm in the brains of adult flies (Fig. 6b). This FA-supplemented diet also suppressed the activation of *dAtf4* (Fig. 6c, d) and rescued the eclosion defects of *dMfn* RNAi flies (Fig. 6e). However, *dMfn* RNAi flies raised on an FA-supplemented diet showed no significant difference in their lifespan (Fig. 6f) or locomotor activity (Fig. 6g) as compared to flies on a normal diet.

### Expression of *Nmdmc* rescues the phenotypes of *dMfn* RNAi flies

**Fig. 7:**
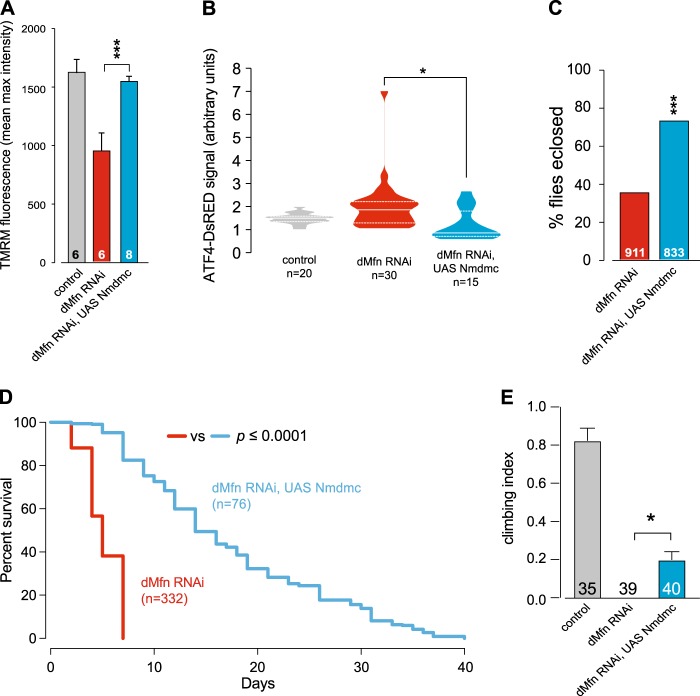
Expression of *Nmdmc* rescues defects associated with downregulation of *dMfn*. Expression of *Nmdmc* rescues (**a**) the loss of Δψm in *dMfn* RNAi flies, mean ± SD; asterisks, one-way ANOVA with Bonferroni’s multiple comparison test and (**b**) the increased expression of *dATF4* in dMfn RNAi flies (asterisks, one-way ANOVA with Dunnett’s multiple comparison test). **c** Expression of *Nmdmc* prevents the eclosion defects in *dMfn* RNAi flies (asterisks, chi-square two-tailed, 95% confidence intervals). **d** Expression of *Nmdmc* increases the lifespan of *dMfn* RNAi flies. The *p*-value is shown for the log-rank Mantel-Cox test. **e** Expression of *Nmdmc* increases locomotor activity of *dMfn* RNAi flies. Flies were tested using a standard climbing assay (mean ± SD; asterisks, one-way ANOVA with Dunnett’s multiple comparison test). Genotypes; (**a**, **c**-**e**) Control: *w; +; daGal4/+*, dMfn RNAi: *w; dMfn RNAi/+; daGal4/+*, dMfn RNAi, UAS Nmdmc: *w; dMfn RNAi/UAS Nmdmc; daGal4/+*, (**b**) Control: *w; ATF4 5’UTR DsRed/+; hhGal4,UASGFP/+*, dMfn RNAi: *w; ATF4.5’UTR* > *dsRed/dMfn RNAi; hhGal4,UASGFP/+*, dMfn RNAi, UAS Nmdmc: *w; ATF4.5’UTR* > *dsRed/dMfn RNAi, UAS Nmdmc; hhGal4,UASGFP/+*. Dataset in A, labelled dMfn RNAi, is also used in Fig. 6b, labelled dMfn RNAi (NF)

Genes involved in FA metabolism, such as *Nmdmc*, comprise a branch of mitochondrial retrograde signalling under the control of the *dAtf4* transcription factor. We therefore tested the effect of enhancing the expression of *Nmdmc* in *dMfn* RNAi flies. Increasing the expression of *Nmdmc* rescued mitochondrial function in neurons (Fig. 7a), suppressed the activation of *dAtf4* (Fig. 7b) and decreased the eclosion defects of *dMfn* RNAi adult flies (Fig. 7c). We also observed that the expression of *Nmdmc* increased the lifespan (Fig. 7d) and decreased the locomotor deficit (Fig. 7e) of *dMfn* RNAi flies. Taken together, these results indicate that the expression of this gene, involved in FA metabolism, has a protective role upon loss of *dMfn*.

## Discussion

Mitochondrial dynamics are coordinated by division and fusion. Fusion is coordinated by mitofusins, nuclear encoded mitochondrial transmembrane GTPases. Mitofusins act by promoting the fusion of the mitochondrial outer membrane (reviewed in^18^). Flies contain two genes coding for mitofusins, with *dMfn* being the only mitofusin expressed in the somatic tissues of adults. Here, we show that the reduction of *dMfn* in adult tissues leads to a fragmentation of mitochondrial cristae, compromising mitochondrial function and transport. Even though it was previously reported that the deletion of *Mfn2* in cultured mouse cells increases mitochondrial mass^19^, we did not detect any alterations of mitochondrial mass after the in vivo depletion of *dMfn*. Our data also suggest that the observed fragmentation of mitochondrial cristae upon loss of *dMfn* is not associated with loss of mitochondria since the overall mitochondrial mass is not altered.

The loss of Δψm and activation of *dAtf4* in flies depleted for *dMf*n could be reversed with dietary supplementation with FA, suggesting that defective mitochondria can act as upstream activators of ER stress pathways. This backs our previous work showing that mitochondrial defects activate ER stress in *pink1* or *parkin* mutant flies^5^.

The climbing defects and decreased lifespan observed in *dMfn* RNAi flies could be improved by the expression of *Nmdmc*, an enzyme involved in FA metabolism, but not by dietary supplementation with FA. Since the intracellular levels of FA intermediates were not measured in our study, we reason that flies lacking *dMfn* might have a decreased food uptake or, alternatively, defects in the intestinal absorption of FA. This is in line with a previous study reporting that mitochondrial fragmentation triggered by the adult expression of Drp1, a protein that promotes mitochondrial fission, compromises the integrity of the fly intestinal barrier^20^. This, in turn, might explain why our pharmacological intervention was less efficient than a genetic enhancement approach.

A therapeutic approach to treat Charcot-Marie-Tooth disease type 2 by manipulating mitofusin conformations has been proposed recently^21^. Our data show that enhancement of FA metabolism might also be a viable approach to treat Charcot-Marie-Tooth disease type 2 and other diseases linked to mitochondrial dysfunction.

## Methods

### Genetics and *Drosophila* strains

Fly stocks and crosses were maintained on standard cornmeal agar media at 25 °C. The strains used were *da*GAL4, *w*^*1118*^, *elav*GAL4*, UASmitoGFP* (Bloomington Stock Centre), RNAi line *dMfn* (ID: 105261,Vienna Drosophila RNAi Center), UAS Nmdmc as previously described^13^, *hhGal4, UASGFP* (kind gift from H. Steller, Rockefeller University, New York, USA), *dpr*Gal4 (kind gift from S. Bullocks, MRC Laboratory of Molecular Biology, Cambridge, UK) and *dATF4.5′UTR* > *dsRed* reporter (kind gift from K. Kang and M-J. Kang, University of Ulsan College of Medicine, Seoul, Republic of Korea). All experiments on adult flies were performed using only males.

### RNA extraction and quantitative real-time RT-PCR

Total RNA was extracted using TRIzol (Ambion) and quantified by spectrophotometric analysis. Quantitative real-time PCR with reverse transcription (qRT-PCR) was performed on a real-time cycler (Applied Biosystems 7500 Fast Real-Time PCR Systems) using the SensiFAST SYBR Lo-ROX one-Step Kit (Bioline). Gene-specific primers were obtained from QIAGEN (QuantiTect Primer Assays) for the following genes: *dMfn* (QT00499205), *Nmdmc* (dm: QT00503153), *Shmt2* (dm: QT00498904). Gene-specific primer *rp49* (forward, TGTCCTTCCAGCTTCAAGATGACCATC; reverse, CTTGGGCTTGCGCCATTTGTG) was obtained from Sigma and used as a housekeeping gene.

### Protein extraction and western blotting

Protein extracts from whole flies were prepared by grinding flies in lysis buffer (100 mM KCl, 20 mM Hepes at pH 7.5, 5% (v/v) glycerol, 10 mM EDTA, 0.1% (v/v) Triton X-100, 10 mM DTT, (1 $\mu g/$mL leupeptin, 1 $\mu g/$mL antipain, 1 $\mu g/$mL chymostatin and 1 $\mu g/$mL pepstatin). The suspensions were cleared by centrifugation at 21,000 × *g* for 10 min at 4 °C and protein concentrations of the supernatants were measured using the Bradford assay (Bio-Rad). All supernatants were mixed with 4 × LDS loading buffer. For SDS–PAGE, equivalent amounts of proteins were resolved on 10% Precast Gels (Invitrogen) and transferred onto PVDF membranes (Millipore). The membranes were blocked in TBS (0.15 M NaCl and 10 mM Tris-HCl, pH 7.5) containing 10% (w/v) dried non-fat milk for 1 h at room temperature, then probed with the primary antibodies (anti-α-tubulin, Sigma, T6074) or anti-dMfn (a gift from A. Whitworth, MRC, Mitochondrial Biology Unit, University of Cambridge, Cambridge, UK), before being incubated with the appropriate HRP-conjugated secondary antibody. Antibody complexes were visualised by Pierce enhanced chemiluminescence (ECL).

### Climbing assay

Climbing assays were performed as previously described^22^ using a counter-current apparatus equipped with six chambers. A total of 15–20 male 3-day-old flies were placed into the first chamber, tapped to the bottom, and then given 20 s to climb a distance of 10 cm. The flies that successfully climbed 10 cm or beyond within 20 s were then shifted to a new chamber, and both sets of flies were given another opportunity to climb the 10 cm distance. This procedure was repeated a total of five times. After five trials, the number of flies in each chamber was counted. A video demonstrating this technique can be found at https://youtu.be/vmR6s_WAXgc. The climbing index was measured using a weighted average approach with the following formula:

$$\frac{{\left( {0 \ast {\mathrm{n}}0} \right) + \left( {1 \ast {\mathrm{n}}1} \right) + \left( {2 \ast {\mathrm{n}}2} \right) + \left( {3 \ast {\mathrm{n}}3} \right) + \left( {4 \ast {\mathrm{n}}4} \right) + \left( {5 \ast {\mathrm{n}}5} \right)}}{{5 \ast {\mathrm{SUM}}\left( {{\mathrm{n}}0:{\mathrm{n}}5} \right)}}$$

In this formula, *n*0 corresponds to the number of flies that failed the first trial, and *n*1 through *n*5 are the numbers of flies that successfully passed each successive trial. At least 100 flies were used for each genotype tested.

### Lifespan analysis

Groups of 15 newly eclosed males of each genotype were placed into separate vials with food and maintained at 25 °C. The flies were transferred to vials containing fresh food every two to three days, and the number of dead flies was recorded. The data are presented as Kaplan–Meier survival distributions, and the significance was determined by the log-rank test.

### Dietary supplements

Folic acid (Sigma, F7876) was incorporated into the fly food at a final concentration of 4 mM. The animals were treated with FA throughout development. Adult flies were maintained on FA-containing food throughout their lifespan, and they were transferred to vials with fresh food every two to three days.

### Microscopy-based assessment of mitochondrial function and length

Measurement of Δψm in brains of 3-day-old flies was performed using tetramethylrhodamine (TMRM) as previously described^17^. Briefly, adult fly brains were loaded with 40 nM TMRM in loading buffer (10 mM HEPES, pH 7.35, 156 mM NaCl, 3 mM KCl, 2 mM MgSO_4_, 1.25 mM KH_2_PO_4_, 2 mM CaCl_2_ and 10 mM glucose) for 40 min at room temperature, and the dye was present during the experiment. In this experiment, TMRM was used in the redistribution mode to assess Δψm, and therefore, a reduction in TMRM fluorescence represents mitochondrial depolarisation. Confocal images were obtained using a Zeiss 510 confocal microscope equipped with a 40× oil immersion objective. Illumination intensity was kept to a minimum (at 0.1–0.2% of laser output) to avoid phototoxicity, and the pinhole was set to give an optical slice of 2 μm. Fluorescence was quantified by exciting TMRM using the 565 nm laser and measured above 580 nm. Z-stacks of 5 fields of 300 μm^2^ each per brain were acquired, and the mean maximal fluorescence intensity was measured for each group.

Mitochondrial length was quantitated in mechanosensory axons in the ventral nerve cord (VNC) from third-instar larvae. The VNC was dissected in PBS, transferred to a drop of PBS as mounting medium on glass slides, covered with a coverslip and imaged on a Zeiss LSM510 confocal microscope. Mitochondrial length was calculated using the “segmented line” tools in ImageJ to measure the length of mitoGFP-positive mitochondria across their largest dimension. *Z*-projection of 10 μm-thick stacks was used to follow the mitochondrial 3D distribution and measure lengths more accurately.

### Citrate synthase assay

Citrate synthase activity was measured using a protocol adapted from the Citrate Synthase Assay kit (CS070 SIGMA). Ten male flies (3-days-old) were homogenised in lysis buffer (100 mM KCl, 20 mM Hepes at pH 7.5, 5% (v/v) glycerol, 10 mM EDTA, 0.1% (v/v) Triton X-100, 10 mM DTT, 1 μg/mL leupeptin, 1 μg/mL antipain, 1 μg/mL chymostatin and 1 μg/mL pepstatin). The suspensions were cleared twice by centrifugation at 2,000 × *g* for 15 s at 4 °C, and the protein concentrations were determined by Bradford assay (Bio-Rad). Sample volume was resuspended in reaction buffer (75 mM Tris-HCl pH 8, 100 μM DTNB, 0.1% Triton, 350 μg/ml, 0.5 mM Oxalacetate) and absorbance was measured at 412 nm for 2 min using M200PRO plate reader (TECAN, Switzerland). Absorbance values were plotted against time (min) for each reaction. Changes in absorbance (ΔA412/minute) were used to calculate the citrate synthase activity using the following equation: units (mmole/ml/min) = (ΔA412/min × V(ml)/ ε^mM^ × L(cm) × V_enz_ (ml). V(ml) = reaction volume, *ε*^mM^ = 13.6 mM^−1^ cm^−1^, V_enz_ (ml) = volume of sample. Units of citrate synthase activity were normalised to protein concentration (mg/ml).

### ATP assays

Five male flies (3-days-old) were homogenised in 100 μL of 6 M guanidine-HCl in extraction buffer (100 mM Tris and 4 mM EDTA, pH 7.8) to inhibit ATPases. Homogenised samples were subjected to rapid freezing in liquid nitrogen followed by boiling for 5 min. Samples were then cleared by centrifugation and the supernatant was diluted (1/50) with extraction buffer and mixed with luminescent solution (CellTiter-Glo Luminescent Cell Viability Assay, Promega). The luminescence was measured on an infinitive M200PRO plate reader (TECAN, Switzerland). The relative ATP levels were calculated by dividing the luminescence by the total protein concentration, which was determined by the Bradford method.

### Analysis of ATF4 activation in *Drosophila* wing discs

To analyse ATF4 activation, ATF4.5′UTR > dsRed reporter larvae with the expression of GFP in the posterior compartment of wing discs under the control of *hh*Gal4 were used. Wing discs were dissected in PBS and imaged on a Zeiss LSM510 confocal microscope. The 15 μm-thick stacks were acquired and maximum projection of DsRed signal was measured by using ImageJ software.

### Measurement of mitochondrial ROS in *Drosophila* adult brain

*Drosophila* adult brains were dissected in PBS and incubated with 5 μM MitoSOX^TM^ Red mitochondrial superoxide indicator (M36008, Molecular Probes) for 30 min. After incubation brains were washed with PBS for 10 min and imaged on a Zeiss LSM510 confocal microscope. The 100 μm-thick stacks were acquired and used to measure the MitoSOX signal using ImageJ software.

### Analysis of mitochondrial speed

Axonal mitochondrial speeds were measured as previously described^23,24^. The 2–5-day-old flies expressing GFP under the *dpr* driver were anaesthetised with CO_2_ and enclosed in a custom-built chamber formed by cover glasses where the body of the fly was placed ventral side up and wings were positioned under small coverslips and covered with a drop of halocarbon oil. Imaging of the axonal wing nerve was performed using a Zeiss LSM510 confocal microscope with a 100× oil immersion objective. Mitochondrial movements were quantified using ImageJ. *Z*-projection was performed and anterograde and retrograde mitochondrial movements were scored following the direction of movement as previously reported. Anterograde runs towards the fly thorax and retrograde to the cell body of the neuron^24^. Transported mitochondria were manually tracked across the frames and speeds calculated dividing the total distance by the time. Data were plotted using Prism (GraphPad).

### Electron microscopy

For transmission electron microscopy, adult fly brains were fixed overnight in 0.1 M sodium cacodylate buffer (pH 7.4) containing 2% paraformaldehyde, 2.5% glutaraldehyde and 0.1% Tween-20. Then, the samples were post-fixed for 1 h at room temperature in a solution containing 1% osmium tetroxide and 1% potassium ferrocyanide. After fixation, the samples were stained en bloc with 5% aqueous uranyl acetate overnight at room temperature; then, they were dehydrated via a series of ethanol washes and embedded in TAAB epoxy resin (TAAB Laboratories Equipment Ltd., Aldermaston, UK). Semi-thin sections were stained with toluidine blue, and areas of the sections were selected for ultramicrotomy. Ultrathin sections were stained with lead citrate and imaged using a MegaView 3 digital camera and iTEM software (Olympus Soft Imaging Solutions GmbH, Münster, Germany) with a Jeol 100-CXII electron microscope (Jeol UK Ltd., Welwyn Garden City, UK).

### Microarray acquisition and analysis

RNA was prepared from 3-day-old male adult flies (8 samples in total, 4 replicates for each genotype). The *dMfn* RNAi was driven by *da*GAL4. The RNA quality was confirmed using an Agilent 2100 Bioanalyzer (Agilent Technologies, CA, USA). Detailed experimental protocols and raw data were deposited in ArrayExpress under accession E-MTAB-6579. Differential expression was analysed using the Partek Genomics Suite (Partek Inc. Missouri, USA). Pathway enrichments were calculated using a pathway ANOVA statistical model in Partek Pathway (Partek Inc. Missouri, USA).

### Respirometry

Mitochondrial respiration in 3-day-old flies was assayed at 37^◦^C by high-resolution respirometry as previously described^25^. The OROBOROS Oxygraph DatLab software package (OROBOROS, Innsbruck, Austria) was used for data acquisition (2 s time intervals) and analysis, including calculation of the time derivative of the oxygen concentration and signal deconvolution dependent on the response time of the oxygen sensor, with correction for instrumental background oxygen flux. Respiration was assessed by homogenising two flies using a pestle in MiR05 respiration buffer (20 mM HEPES, 10 mM KH_2_PO_4_, 110 mM sucrose, 20 mM taurine, 60 mM K-lactobionate, 0.5 mM EGTA, 3 mM MgCl_2_, and 1 g/l fatty acid-free BSA). Coupled state 3 respiration for complex I was assayed in MiR05 respiration buffer in the presence of 2 mM malate, 10 mM glutamate and 5 mM ADP.

### Statistical analyses

Descriptive and inferential statistical analyses were performed using GraphPad Prism 8 (www.graphpad.com). The data are presented as the mean value, and the error bar indicates ± SD or ± SEM (as indicated). In the combined violin and box plots, the median is shown with a solid white line and the quartiles are represented by the dashed white lines. The number of biological replicates per experimental variable (*n*) is indicated in either the respective figure or figure legend. Significance is indicated as **** for *p* < 0.0001; *** for *p* < 0.001; ** for *p* < 0.01 and * for *p* < 0.05. The investigators gathering quantitative data on the biological samples were not blinded to the sample identities at the time of analysis. No specific randomisation strategies were employed when the biological replicates were assigned to the treatment groups.

### Digital image processing

Western blot images were acquired as uncompressed, bitmapped digital images (TIFF format). The images were processed using Adobe Photoshop, employing established scientific imaging workflows^26^.

## Supplementary information

Table 1

Table 2

Table 3

Supplementary figure legends
