# Supplementary material for: Enhancing folic acid metabolism suppresses defects associated with loss of Drosophila mitofusin
Source: Cell Death Dis. 2019 Mar 25;10(4):288. doi: 10.1038/s41419-019-1496-2 (PMC6433915; doi:10.1038/s41419-019-1496-2)
Supplement: Supplementary file 4 — Supplementary figure legends [file 41419_2019_1496_MOESM4_ESM.docx]

## SUPPLEMENTATY INFORMATION

## SUPPLEMENTARY TABLE LEGENDS

### Supplementary Table 1. Transcriptional profile of adult flies following RNAi-mediated suppression of *dMfn*.

A list of the 16,322 transcripts detected in adult flies and analysed using the Affymetrix Gene 1.1 ST Array Platform.

### Supplementary Table 2. Transcripts positively regulated by the RNAi-mediate suppression of *dMfn*.

The 275 transcripts listed show a fold-change ≥ 1.6 and an FDR (step-up) ≤ 0.05 in *dMfn* RNAi adult flies.

### Supplementary Table 3. Pathway analysis of transcripts positively regulated by the RNAi-mediated suppression of *dMfn*.

Pathway analysis was performed using Partek Pathway for the 275 transcripts showing a fold-change ≥ 1.6 and an FDR (using a step-up procedure) ≤ 0.05 in *dMfn* RNAi adult flies.
